# Cross-kingdom patterns of alternative splicing and splice recognition

**DOI:** 10.1186/gb-2008-9-3-r50

**Published:** 2008-03-05

**Authors:** Abigail M McGuire, Matthew D Pearson, Daniel E Neafsey, James E Galagan

**Affiliations:** 1 The Broad Institute of MIT and Harvard, Cambridge Center, Cambridge, MA 02142, USA

## Abstract

**Background:**

Variations in transcript splicing can reveal how eukaryotes recognize intronic splice sites. Retained introns (RIs) commonly appear when the intron definition (ID) mechanism of splice site recognition inconsistently identifies intron-exon boundaries, and cassette exons (CEs) are often caused by variable recognition of splice junctions by the exon definition (ED) mechanism. We have performed a comprehensive survey of alternative splicing across 42 eukaryotes to gain insight into how spliceosomal introns are recognized.

**Results:**

All eukaryotes we studied exhibit RIs, which appear more frequently than previously thought. CEs are also present in all kingdoms and most of the organisms in our analysis. We observe that the ratio of CEs to RIs varies substantially among kingdoms, while the ratio of competing 3' acceptor and competing 5' donor sites remains nearly constant. In addition, we find the ratio of CEs to RIs in each organism correlates with the length of its introns. In all 14 fungi we examined, as well as in most of the 9 protists, RIs far outnumber CEs. This differs from the trend seen in 13 multicellular animals, where CEs occur much more frequently than RIs. The six plants we analyzed exhibit intermediate proportions of CEs and RIs.

**Conclusion:**

Our results suggest that most extant eukaryotes are capable of recognizing splice sites via both ID and ED, although ED is most common in multicellular animals and ID predominates in fungi and most protists.

## Abstract 2

A comprehensive survey of alternate splicing across 42 eukaryotes so as to gain insight into how spliceosomal introns are recognized.

## Background

Intron splicing occurs in all domains of life, but the splicing methods employed and the frequencies of splicing vary among organisms. Bacteria and archaea lack the spliceosomal pathway and splice infrequently via self-splicing introns. Among unicellular eukaryotes, there is substantial range in splicing frequency [1,2]. Many early-branching eukaryotes, including the protists *Giardia*, *Cryptosporidia*, *Trypanosoma*, *Entamoeba*, and *Trichomonas*, have few or no introns. Only 5% of genes are spliced in *Saccharomyces cerevisiae *[3], a yeast, while the average number of introns per gene among other fungi is generally low (with a few noteworthy exceptions). Their average intron density ranges from just over one in *Schizosaccharomyces pombe *to approximately five in *Cryptococcus neoformans *[4]. Protists have similarly low rates of splicing. In contrast, multicellular animals often have large numbers of introns (over seven per gene in vertebrates), while plants have intermediate numbers of introns (approximately four per gene in *Oryza sativa *and *Arabidopsis thaliana*).

The number of introns and recognized splice sites may vary between individual mRNA transcripts of a single gene, giving rise to the phenomena of splice variation and alternative splicing. In this paper we use 'splice variation' to describe any difference in intron processing, reserving the term 'alternative splicing' for splice variation that is regulated and functionally significant. Observed splice variation is a combination of programmed alternative splicing events and splicing errors. Functional alternative splicing may result from various causes, including ontogenic changes and environmental stimuli. As the number of genes in an organism is not well correlated with its complexity, alternative splicing may provide an additional layer of regulation that permits greater complexity in higher organisms [5]. Multicellular organisms may generate different splice forms of the same gene in different tissues, or even within different cells in the same tissue [5,6]. More recently, it has also been demonstrated that alternative splicing can vary between individuals in a heritable manner [7].

Splice variants can be divided into four broad categories: retained introns (RIs), cassette exons (CEs), competing 5' splice sites, and competing 3' splice sites. CEs are the predominant form of splice variation in multicellular eukaryotes [8-10], whereas RIs are more frequent in multicellular plants such as *A. thaliana *and *O. sativa *[11-13], as well as the fungus *Cryptococcus *and in yeast [14-17].

**Figure 1:**
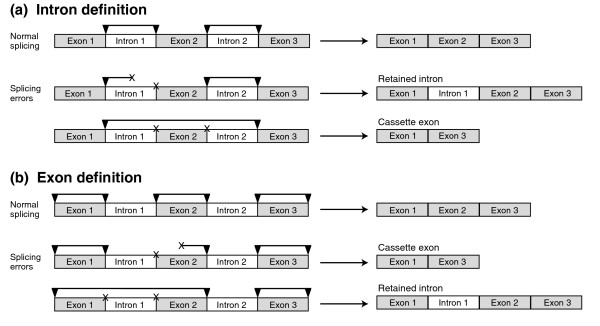
Effects of splicing errors under the intron definition (ID) and exon definition (ED) models. Arrowheads connected by horizontal bars illustrate the paired recognition of splice sites. **(a) **When splice sites are recognized in pairs across introns by ID, an error at a single splice site (marked 'x') prevents the removal of an intron, leading to a RI. Under ID, two adjacent splice sites must be mis-spliced, and the splicing machinery must operate over a greater distance, to generate a CE. **(b) **In the ED model, splice sites are recognized in pairs across exons. An error at a single splice site results in a CE. Obtaining an RI via ED requires coordinated mis-recognition of two adjacent splice sites over a longer distance. Observed RIs can be parsimoniously explained by ID-mediated splicing, while observed CEs likely indicate splicing via ED.

The profile of splice variants in a given organism is likely influenced by the mechanisms it uses to identify and process splice sites. In eukaryotes, it has been proposed that the spliceosome recognizes splice sites in pairs, either across the intron (intron definition (ID)) or across the exon (exon definition (ED)) [9]. In ID, splice sites on either side of an intron are recognized as a unit, while in ED, splice sites on either side of an exon are recognized as a unit. Experiments in both yeast and *Drosophila *have shown that when splice sites are presumably recognized by ID, mutating a single splice site disrupts splicing of the intron adjacent to the mutation. This leads to an RI, but has no effect on the splicing of nearby introns [17,18] (Figure 1). In contrast, when splice sites are presumably recognized by ED, mutating a single splice site affects not only the splicing of the intron adjacent to the mutation, but also the intron on the other side of the exon adjacent to the mutation. This causes cassette exons to be skipped [19,20]. Therefore, it is believed that with ID, splicing errors are more likely to result in RIs, while with ED, splicing errors are more likely to result in CEs. ID and ED are not mutually exclusive; in *Drosophila melanogaster*, ID and ED have been shown to operate within a single mRNA [21].

The method used to recognize splice sites has been associated with restrictions on exon and intron length. Recognition of splice sites with ED appears to constrain exon length [20,22], while recognition with ID limits intron length [18,23]. Fox-Walsh *et al*. [24] suggest that splice site recognition across the intron in *D. melanogaster *ceases at lengths greater than around 200-250 bp. A review of previous studies suggests that phylogenetic trends in exon and intron length may be correlated with the relative occurrence of RIs and CEs and the use of ID or ED for splice junction recognition [16,18,20,23,24]. However, previous results have been limited in their phylogenetic scope.

In this paper, we report a comprehensive survey of splice variants in 42 eukaryotic organisms. Our survey covers a wide phylogenetic range, including 13 multicellular animals, 6 plants, 14 fungi, and 9 protists. We observe variation across major phylogenetic groups in the representation of RIs and CEs among splice variants that is consistent with variation in the mode of splice site recognition (ID or ED) used by these groups. We infer that groups with a high ratio of RIs to CEs (fungi and protists) operate predominantly by ID, while groups with a low ratio of RIs to CEs (multicellular animals) operate predominantly by ED. In organisms with evidence of both RIs and CEs (thus, employing both ID and ED), CEs are shorter than constitutive exons (exons that show no evidence of splice variation), and RIs are shorter than constitutive introns, suggesting that splice mechanisms are closely tied to gene structure.

## Results

To assess splice variation in eukaryotes, we selected 42 organisms with genome assemblies and large numbers of publicly available expressed sequence tags (ESTs; Table 1), spanning the plants, fungi, protists and multicellular animals. We aligned ESTs to genome assemblies and constructed transcript fragments, examined all loci where the EST data indicated two or more overlapping non-compatible transcripts, and labeled every instance of splice variation. Table 2 shows the numbers of ESTs for each organism, as well as the numbers of transcripts and loci constructed. Table 3 lists the splice variants we found. A complete list of the locations of predicted sites of splice variation, as well as control introns and exons that show no splice variation despite high EST coverage, is available on the Broad Institute's ftp site [25].

**Table 1:** Genomes and ESTs used in the analysis

Genome name	Kingdom	Reference	ESTs
*Danio rerio*	Animal	Assembly Zv6, Sanger Institute [66]	Genbank 06/15/07
*Takifugu rubripes*	Animal	[67]	Genbank 06/22/07
*Branchistoma floridae*	Animal	JGI [60] (v1.0)	Genbank 06/22/07
*Ciona savignyi*	Animal	Broad Institute [58] (ci1.0)	Genbank 06/15/07
*Ciona intestinalis*	Animal	[68]	Genbank 06/15/07
*Nematostella vectensis*	Animal	[69]	Genbank 06/22/07
*Strongylocentrotus purpuratus*	Animal	[70]	Genbank 06/27/07
*Drosophila melanogaster*	Animal	[71]	Genbank 06/15/07
*Aedes aegypti*	Animal	[72]	Vectorbase [61,73]
*Anopheles gambiae*.	Animal	[74]	Vectorbase [61,73]
*Apis mellifera*	Animal	[75]	Genbank 06/18/07
*Caenorhabditis elegans*	Animal	[76]	Genbank 02/1/07
*Schistosoma mansoni*	Animal	TIGR/JCVI [59]	Genbank 06/22/07
*Rhizopus oryzae*	Fungi	Broad Institute [58]	Genbank 06/15/07 + Broad Institute [58]
*Cryptococcus neoformans *JEC21	Fungi	[15]	Genbank 06/19/07
*Ustilago maydis*	Fungi	[77]	Genbank 06/15/07
*Schizosaccharomyces pombe*	Fungi	[78]	Genbank 01/14/08
*Saccharomyces cerevisiae*	Fungi	[79]	Genbank 01/14/08
*Neurospora crassa*	Fungi	[80]	Genbank 06/15/07
*Magnaporthe grisea *70-15	Fungi	[81]	Genbank 06/15/07
*Stagnospora nodorum*	Fungi	Broad Institute [58]	Genbank 06/15/07
*Aspergillus flavus *NRRL3357	Fungi	TIGR/JCVI [59], GenBank AAIH00000000	Genbank 06/15/07
*Aspergillus nidulans*	Fungi	[82]	Genbank 06/15/07
*Histoplasma capsulatum*	Fungi	Broad Institute [58]	Genbank 06/15/07
*Coccidioides posadasii*	Fungi	TIGR/JCVI [59]	Genbank 06/15/07
*Coccidioides immitis RS*	Fungi	Broad Institute [58]	Genbank 06/15/07
*Sclerotinia sclerotiorum 1980*	Fungi	Broad Institute [58]	Genbank 06/15/07 + Broad Institute [58]
*Dictyostelium discoideum*	Protist	[83]	Genbank 06/15/07
*Populus trichocarpa*	Plant	[84]	Genbank 06/18/07
*Arabidopsis thaliana*	Plant	[85]	Genbank 06/16/07
*Oryza sativa*	Plant	[86]	Genbank 06/15/07
*Physcomitrella patens*	Plant	JGI [60] (v1.1)	Genbank 06/28/07
*Chlamydomonas reinhardtii*	Plant	JGI [60] (v3.0)	Genbank 06/18/07
*Ostreococcus lucimarinus*	Plant	[87]	JGI [60]
*Phytophthora infestans*	Protist	Broad Institute [58] (C Nusbaum, personal communication)	Genbank 06/15/07
*Phytophthora sojae*	Protist	[88]	Genbank 06/15/07
*Plasmodium yoelii*	Protist	[89]	Genbank 06/15/07
*Plasmodium falciparum 3D7*	Protist	[90]	Genbank 06/19/07
*Paramecium tetraurelia*	Protist	[91]	Genbank 06/15/07
*Tetrahymena thermophila*	Protist	[92]	Genbank 06/19/07
*Entamoeba hystolitica*	Protist	[4]	Genbank 06/18/07
*Phaeodactylum tricornutum*	Protist	JGI [60]	Genbank 06/28/07

**Table 2:** Numbers of ESTs, transcripts, and loci

	Kingdom	Total ESTs*	Spliced. Filtered ESTs^†^	Transcripts^‡^	Loci^§^
*D. rerio*	Animal	1350,105	556,175	76,066	33,338
*T. rubripes*	Animal	26,069	14,197	5,890	4,988
*B. floridae*	Animal	277,538	57,883	15,784	12,054
*C. savignyi*	Animal	84,302	24,196	7,027	8,382
*C. intestinalis*	Animal	686,396	334,137	31,794	15,108
*N. vectensis*	Animal	16,619	6,206	4,609	4,294
*S. purpuratus*	Animal	141,833	24,770	23,256	20,773
*D. melanogaster*	Animal	532,557	242,235	25,241	14,965
*A. aegypti*	Animal	303,409	120,523	15,878	11,389
*A. gambiae*	Animal	216,617	95,607	13,511	9,835
*A. mellifera*	Animal	78085	32,860	9,581	7,800
*C. elegans*	Animal	346,064	219,812	28,438	21,304
*S. mansoni*	Animal	158,841	55,392	14,494	10,137
*R. oryzae*	Fungi	25,393	12,238	3,263	3,052
*C. neoformans*	Fungi	59,041	46,693	8,173	6,361
*U. maydis*	Fungi	39,308	11,236	1,289	1,109
*S. pombe*	Fungi	5,574	843	274	269
*S. cerevisiae*	Fungi	32,653	2,251	259	245
*N. crassa*	Fungi	28,089	7,577	1,571	1,495
*M. grisea*	Fungi	53,102	14,563	3,229	2,795
*S. nodorum*	Fungi	15,973	5,925	1,637	1,537
*A. flavus*	Fungi	20,371	8,495	3,004	2,772
*A. nidulans*	Fungi	16,848	5,499	2,240	2,090
*H. capsulatum*	Fungi	26,389	2,334	850	950
*C. posadasii*	Fungi	54,217	30,604	6,769	5,296
*C. immitis*	Fungi	65,754	32,162	6,484	5,133
*S. sclerotiorum*	Fungi	65,884	30,203	4,314	3,704
*D. discoideum*	Protist	155,032	46,116	4,687	4,246
*P. trichocarpa*	Plant	89,943	38,299	11,462	9,377
*A. thaliana*	Plant	1,276,692	350,380	35,856	23,412
*O. sativa*	Plant	977,774	374,397	43,265	25,610
*P. patens*	Plant	194,822	106,309	21,962	15,402
*C. reinhardtii*	Plant	167,641	72,903	11,353	8,514
*O. lucimarinus*	Plant	19,200	1,043	328	304
*P. infestans*	Protist	94,091	17,381	4,762	4,104
*P. sojae*	Protist	28,357	7,418	2,125	2,012
*P. yoelii*	Protist	13,925	2,863	1,019	931
*P. falciparum*	Protist	21,349	3,928	1,417	1,219
*P. tetraurelia*	Protist	86,070	44,772	11,423	10,258
*T. thermophila*	Protist	56,543	21,073	6,035	5,540
*E. histolytica*	Protist	20,404	599	174	166
*P. tricornutum*	Protist	89,139	14,576	3,325	2,937

*Number of raw ESTs before filtering. ^†^Number of ESTs aligned after applying our set of filters, containing at least one splice site (see Materials and methods). ^‡^Number of 'transcripts' constructed from the ESTs. ^§^Number of 'loci' (overlapping clusters of transcripts) (genes with 1+ splice site).

**Table 3:** Types of splice variants observed

				Retained introns				
							
	Kingdom	No. of splice variants	Cassette exons	Discarding unspliced ESTs	Keeping unspliced ESTs*	Competing 5' sites	Competing 3' sites	$\frac{CE}{RI+CE}$ (CE fraction)
*D. rerio*	Animal	6,137	2,475	1,176	2,324	931	1,555	0.68
*T. rubripes*	Animal	201	52	47	79	50	52	0.53
*B. floridae*	Animal	812	516	26	123	121	149	0.95
*C. savignyi*	Animal	181	93	18	64	22	48	0.84
*C. intestinalis*	Animal	2,779	834	907	2,084	394	644	0.48
*N. vectensis*	Animal	108	54	8	33	21	25	0.87
*S. purpuratus*	Animal	284	147	13	50	63	61	0.92
*D. melanogaster*	Animal	2,163	453	712	2,343	442	556	0.39
*A. aegypti*	Animal	983	280	284	606	186	233	0.50
*A. gambiae*	Animal	882	207	283	635	190	202	0.42
*A. mellifera*	Animal	561	153	124	338	135	149	0.55
*C. elegans*	Animal	1,589	361	452	828	285	491	0.44
*S. mansoni*	Animal	1,236	235	599	974	143	259	0.28
*R. oryzae*	Fungi	47	0	30	55	5	12	0
*C. neoformans*	Fungi	1,091	18	768	1,117	101	204	0.02
*U. maydis*	Fungi	85	8	31	105	16	30	0.21
*S. pombe*	Fungi	3	0	3	13	0	0	0
*S. cerevisiae*	Fungi	9	0	2	42	3	4	0
*N. crassa*	Fungi	20	2	8	30	5	5	0.20
*M. grisea*	Fungi	151	5	86	194	24	36	0.05
*S. nodorum*	Fungi	36	2	21	37	5	8	0.09
*A. flavus*	Fungi	162	3	124	302	13	22	0.02
*A. nidulans*	Fungi	100	1	74	216	8	17	0.01
*H. capsulatum*	Fungi	50	1	33	65	6	10	0.03
*C. posadasii*	Fungi	950	15	648	1,259	120	167	0.02
*C. immitis*	Fungi	861	8	542	1,035	131	180	0.01
*S. sclerotiorum*	Fungi	323	9	210	419	49	55	0.04
*D. discoideum*	Protist	107	6	33	98	29	39	0.15
*P. trichocarpa*	Plant	664	144	215	392	103	202	0.40
*A. thaliana*	Plant	3,255	251	1,260	2,609	547	1,197	0.17
*O. sativa*	Plant	3,893	450	1,339	3,076	706	1,398	0.25
*P. patens*	Plant	2,068	249	534	1,156	558	727	0.32
*C. reinhardtii*	Plant	490	60	211	520	86	133	0.22
*O. lucimarinus*	Plant	17	0	13	60	2	2	0
*P. infestans*	Protist	406	6	262	564	60	78	0.02
*P. sojae*	Protist	66	1	32	87	15	18	0.03
*P. yoelii*	Protist	38	1	22	92	6	9	0.04
*P. falciparum*	Protist	77	9	34	75	19	15	0.21
*P. tetraurelia*	Protist	535	0	407	786	35	93	0
*T. thermophila*	Protist	282	6	202	423	30	44	0.03
*E. histolytica*	Protist	7	0	6	24	0	1	0
*P. tricornutum*	Protist	215	0	126	643	27	62	0

*For comparison, we include the total number of RIs predicted when unspliced ESTs are not discarded. Discarding unspliced ESTs primarily affects the number of predicted RIs. Complete results for all forms of alternative splicing, when unspliced ESTs are not discarded, are included in Additional data file 5.

### All eukaryotes exhibit splice variation

We found that splice variation is present in all organisms we analyzed. Every eukaryote we studied exhibited RIs, and almost every organism exhibited competing 5' splice sites, competing 3' splice sites, and CEs. Several organisms showed zero or very few CEs or competing splice sites due to having only a small EST library or a small overall number of predicted splice variants (for example, *Histoplasma capsulatum*, *Rhizopus oryzae*, *Entamoeba histolytica*), or a small number of introns (for example, *S. cerevisiae*). We also found no CEs in *Paramecium tetraurelia*, despite a large EST library and numerous predicted splice variants. However, *P. tetraurelia *is unusual in that it has extraordinarily short introns (25 bp on average). As CEs are usually associated with longer introns and shorter exons, it is possible that this organism's gene structure renders CEs impossible.

**Figure 2:**
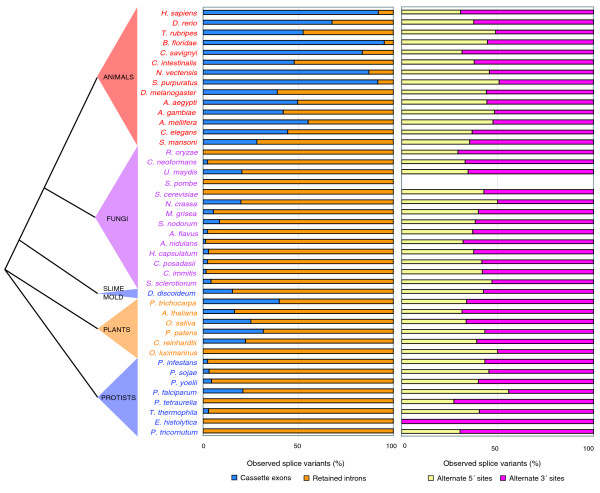
Frequencies of different forms of splice variation, arranged by phylogenetic group. The two bar charts show the relative frequencies of each type of splice variation. The ratio of CEs to RIs is shown in the chart on the left, while the one on the right displays competing 5' and competing 3' splice sites. Note that the CE/RI ratio shows wide variation among kingdoms while the ratio of competing 5' to competing 3' splice sites is remarkably consistent. A high-level overview of the phylogenetic tree is shown on the far left, and the organisms' names are colored according to their phylogenetic grouping. To see all four forms of splice variation on a single bar plot, see Additional data file 1. The data for *H. sapiens *was taken from a previous study [8].

Figure 2 illustrates the relative proportions of the four different kinds of splice variants in each organism, along with previously published data for human for comparison [8]. Our results for *Caenorhabditis elegans*, *D. melanogaster*, *A. thaliana*, and *O. sativa *confirmed those of previous studies [10,12,13].

The ratio of competing 3' splice sites to competing 5' splice sites was fairly constant, with more competing 3' splice sites than competing 5' splice sites in almost every case (Table 3). This is consistent with results of previous studies of splice variation, including the nine organisms in the Altextron database [10] and several other organisms [8,9,12]. When we combine the data from all the organisms in our analysis, there are 1.7 times more competing 3' splice sites than competing 5' splice sites. Interestingly, Zavolan *et al*. [26] found that competing 3' splice sites are more likely to preserve the reading frame than competing 5' splice sites.

In contrast to the uniform ratio of competing splice sites, the ratio of CEs to RIs varies widely between organisms. We found the ratio $\frac{CE}{RI+CE}$ (which we will refer to as the CE fraction) to be a useful metric for summarizing the pattern of these splice variants. The CE fraction is listed in Table 3 and illustrated in Figure 2 for each organism.

### CE and RI prevalence vary by kingdom and by intron length

Major eukaryotic groups (animals, plants, fungi, and protists) exhibit very divergent CE fractions (Figure 2). We found that RIs are the dominant form of splice variation in fungi and most protists, while CEs are the dominant form in multicellular animals. Plants have intermediate proportions of CEs and RIs. The difference in the proportions of RIs and CEs between the group of animals and the group consisting of all fungi and protists is highly statistically significant (*p *< 1e-10 by Fisher's exact test).

The 13 multicellular animals in our analysis have 1.3 times more CEs than RIs, which corresponds to an overall average CE fraction of 55%. CE fractions for these organisms range from 28% for the flatworm *Schistosoma mansoni *to 95% for the chordate *Branchiostoma floridae*. The four insects we studied have an average CE fraction of 44%. Moreover, variation in the CE fraction within chordates appears to be associated with genome size, serving as a partial control for phylogenetic effects. *Takifugu rubripes *has a more compact genome than the other two chordates in our analysis (*Danio rerio *and *B. floridae*), and has a correspondingly lower CE fraction (53%) than they do (*D. rerio *has a CE fraction of 68%; *B. floridae*, 95%).

In contrast, among the unicellular fungi and protists, we see very few CEs and an overwhelming preference for RIs. Overall, fungi and protists have 37 times more RIs than CEs (an average CE fraction of 3%). This preference for intron retention is consistent with previous reports on baker's yeast and fission yeast [17,27], although our kingdom-wide sampling indicates RI predominance is not limited to the highly derived yeasts. RIs also dominate in *C. neoformans *[15], a member of a group of intron-rich fungi, indicating that RI dominance in fungi is not coupled with intron density.

Plants in turn appear intermediate between animals and fungi in their relative amounts of CEs and RIs. We examined four multicellular plants: *A. thaliana*, *O. sativa *(rice), *Populus trichocarpa *(cottonwood), and *Physcomitrella patens *(a moss), as well as two unicellular green algae (*Chlamydomonas. reinhardtii *and *Ostreococcus lucimarinus*). Overall, we found 3.1 times more RIs than CEs, with an average CE fraction of 24%, consistent with previous studies in *A. thaliana *and *O. sativa *[11-13]. The unicellular algae *C. reinhardtii *has a CE fraction of 22%, which is closer to the values seen in multicellular plants than other unicellular organisms. However, it has a large genome size for a unicellular organism (118 Mb). In contrast, *O. lucimarinus *is a much simpler unicellular green algae with smaller genome size (13 Mb), minimal cellular organization and no CEs, a genome structure that is more like those of unicellular fungi and protists.

**Figure 3:**
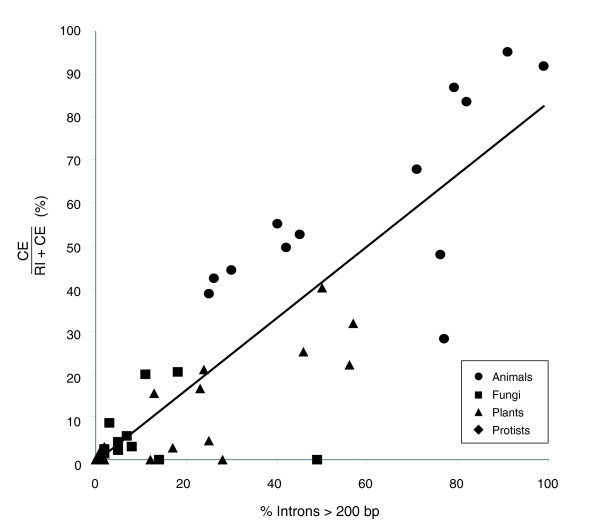
Relationship between long introns and CE fraction. The percentage of long introns (greater than 200 bp) is correlated with the CE fraction ($\frac{CE}{RI+CE}$). The best-fit line is y = 0.84x + 0.00 (R^2 ^= 0.73). In each of four major eukaryotic groups (animals, fungi, plants and protists), species with more long introns display a higher propensity toward CEs.

The observed variation in CE fraction closely parallels variation in intron length. Animals and plants have more long introns (introns greater than 200 bp) than do protists and fungi (Table 4). In Figure 3 we plot the fraction of constitutive introns greater than 200 bp versus the CE fraction, and demonstrate a direct correlation between the presence of long introns and high incidence of CEs (y = 0.84x + 0.00; R^2 ^= 0.73). This correlation also holds within each kingdom (fungi, protists, plants, and animals), providing a phylogenetic control. As discussed below, this correlation is consistent with the hypothesis that splice site recognition differs within these groups.

**Table 4:** Intron and exon lengths for controls and splice variants

	Kingdom	Intron density*	Genome assembly size (Mb)^†^	Average intron length^‡^	Average RI length^§¶^	Average intron length next to CE^¥^	No. of CEs with unambiguous boundaries^#^	% introns >200 bp**	Average exon length^¶††^	Average internal exon length^¶‡‡^	Average CE length^§§^
*D. rerio*	Animal	7.2	1,547	2,940	130	3,485	903	71%	180	132	113
*T. rubripes*	Animal	8.1	393	582	126	641	22	45%	140	119	103
*B. floridae*	Animal	5.7	923	1,283	115	1,424	135	91%	151	124	111
*C. savignyi*	Animal	9.1	164	710	87	595	20	82%	148	130	123
*C. intestinalis*	Animal	7.4	117	457	152	544	327	76%	181	143	122
*N. vectensis*	Animal	4.1	357	823	167	820	26	79%	165	110	90
*S. purpuratus*	Animal		907	1,819	176	2,085	40	99%	174	141	133
*D. melanogaster*	Animal	3.9	120	829	90	1,632	218	25%	279	246	167
*A. aegypti*	Animal	3.1	1,384	4,614	106	7,752	112	42%	309	252	204
*A. gambiae*	Animal	3.2	278	1,154	110	2,905	83	26%	286	236	181
*A. mellifera*	Animal	5.4	454	1,171	107	2,969	98	40%	195	172	153
*C. elegans*	Animal	5.5	100	259	84	497	176	30%	189	181	151
*S. mansoni*	Animal	4.8	381	2,222	42	2,387	127	77%	179	167	130
*R. oryzae*	Fungi	2.3	46	58	66	-	0	1%	201	161	-
*C. neoformans*	Fungi	5.4	19	64	67	95	8	5%	228	179	56
*U. maydis*	Fungi	0.8	20	168	134	288	5	18%	256	143	52
*S. pombe*	Fungi	1.3	13	108	58	-	0	14%	309	102	-
*S. cerevisiae*	Fungi	0.1	12	241	244	-	0	49%	596	46	-
*N. crassa*	Fungi	1.8	39	115	126	171	2	11%	206	142	78
*M. grisea*	Fungi	1.7	42	109	110	103	5	7%	238	160	64
*S. nodorum*	Fungi	1.7	37	70	82	88	2	3%	257	178	17
*A. flavus*	Fungi	2.3	40	76	76	76	1	2%	249	161	90
*A. nidulans*	Fungi	2.8	30	74	87	78	1	2%	204	151	8
*H. capsulatum*	Fungi	2.5	33	112	114	114	1	8%	227	155	25
*C. posadasii*	Fungi	2.2	27	80	87	137	10	2%	395	289	81
*C. immitis*	Fungi	2.5	29	80	83	89	3	2%	349	244	54
*S. sclerotiorum*	Fungi	1.8	38	84	98	71	7	5%	316	213	56
*D. discoideum*	Protist	1.3	34	145	107	252	3	13%	265	231	110
*P. trichocarpa*	Plant	3.1	308	431	140	637	80	50%	204	125	103
*A. thaliana*	Plant	4.9	119	180	118	242	138	23%	201	143	101
*O. sativa*	Plant	4.4	371	462	134	725	299	46%	212	135	116
*P. patens*	Plant	3.8	480	295	188	394	188	57%	220	137	113
*C. reinhardtii*	Plant	7	118	264	148	376	30	56%	172	121	103
*O. lucimarinus*	Plant	0.5	13	157	97	-	0	28%	437	135	-
*P. infestans*	Protist	2	229	76	78	88	5	1%	214	146	92
*P. sojae*	Protist		86	87	107	75	1	2%	213	147	111
*P. yoelii*	Protist	1.2	23	179	151	157	1	25%	229	127	18
*P. falciparum*	Protist	1.8	23	157	119	176	8	24%	189	112	113
*P. tetraurelia*	Protist	2.5	72	25	26	-	0	0%	246	233	-
*T. thermophila*	Protist	3.3	104	132	103	136	3	17%	234	166	83
*E. histolytica*	Protist		23	72	62	-	0	2%	296	168	-
*P. tricornutum*	Protist	0.8	26	128	109	-	0	12%	446	410	-

*(Number of introns in genome/Number of genes in genome), calculated from genome annotations. ^†^The length of the assembly used in our analysis. ^‡^Calculated from constitutive introns in EST alignments. ^§^Average length of RIs seen in our EST alignments. ^¶^See Table S2 in Additional data file 4 for average lengths based on genome annotations. ^¥^Average length of the two introns surrounding each predicted CE in our EST alignments. ^#^Number of CEs considered for 'Average intron length next to CE' in the previous column and in Figure 5. This excludes those CEs where the introns next to the CEs do not have identical boundaries between transcripts with and without that CE. **Fraction of constitutive introns longer than 200 bp. ^††^Calculated from constitutive exons in our EST alignments. ^‡‡^Calculated from constitutive exons in our EST alignments (excluding exons that cannot be CEs, namely, the first and last exons in a gene, and exons in genes without introns). ^§§^Average length of CEs seen in our EST alignments.

### Variably spliced regions exhibit size constraints

**Figure 4:**
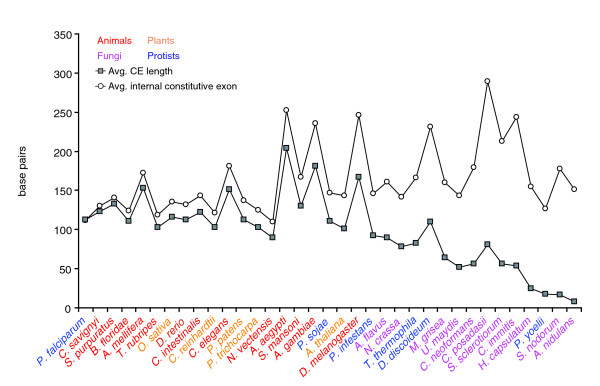
Average lengths of CEs compared to average lengths of internal constitutive exons. Species are sorted by the fractional difference between these two lengths. In organisms where CEs are common (animals and plants) CEs are almost identical in length to constitutive exons, while in species where CEs are rare (fungi and protists) CEs tend to be significantly shorter than constitutive exons. In animals and plants, where ED is common, CEs are spliced by the same process as constitutive exons and these two groups are thus subject to the same length constraints. In organisms that splice primarily by ID, including fungi and protists, the lengths of constitutive exons are not constrained by ED. However, CEs in these organisms are still recognized by ED. Thus, in these species, constitutive exons can grow longer than CEs.

**Figure 5:**
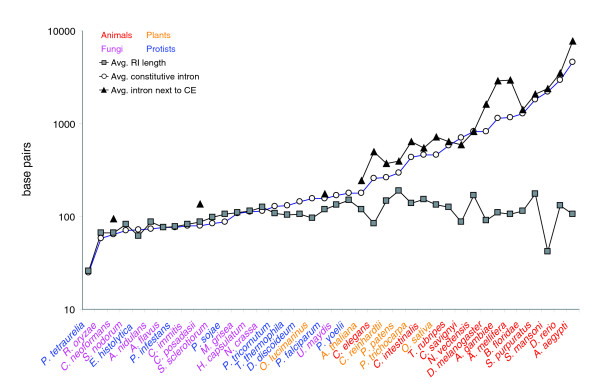
Average lengths of RIs compared with lengths of constitutive introns and introns adjacent to CEs. RI length, which is constrained by ID, is fairly constant across all organisms. In protists and fungi, average RI length is close to that of constitutive introns, because ID is the primary mode of splice site recognition for both groups. In animals, constitutive intron length differs substantially from RI length because most constitutive introns are recognized by ED and are not subject to the same length constraints as RIs, which are recognized by ID. Plants fall between unicellular organisms and animals. Data are shown only for organisms with at least five RIs. For introns next to CEs, data are shown only for organisms with at least eight CEs with unambiguous adjacent intron lengths on both sides. Introns next to CEs are usually longer than constitutive introns, because these introns are recognized by ED and are free from ID length constraints.

As shown in Table 4, variably spliced introns and exons are usually shorter than those in our data set that display no splice variation, in agreement with previous observations [28]. Moreover, these length differences between constitutive and variably spliced introns and exons appear to be associated with the relative frequencies of splice variation via CEs and RIs. In organisms where CEs are rare, such as fungi, CEs tend to be noticeably shorter than internal constitutive exons. However, in organisms with substantial fractions of CEs (animals and multicellular plants) we observe no significant length difference between CEs and internal constitutive exons (Figure 4). Intron retention displays the opposite behavior. In organisms where RIs are uncommon (animals and multicellular plants), RIs tend to be shorter than constitutive introns, while organisms with large numbers of RIs (fungi and protists) show no substantial length difference between RIs and constitutive introns (Figure 5). In general, CEs and RIs both tend to be shorter than their constitutively spliced counterparts, with the length difference most noticeable in organisms in which each splice variant was uncommon.

### Most splice variants are not functional

We next sought to determine the degree to which the observed splice variants reflect programmed alternative splicing versus incomplete splicing or splicing errors. To do so, we examined the impact of observed splice variants on the corresponding open reading frame and resultant protein. We also examined conservation within regions containing splice variants to look for signatures of coding selection.

Previous analyses of splice variants in mammals have focused on the more prevalent CEs. One surprising result from these analyses is the high frequency of CEs that alter reading frame or introduce stop codons [29]. Overall, approximately half of human CEs in coding regions result in frameshifts, while an additional 15% of CEs that do not cause frameshifts introduce in-frame stop codons [29]. A more recent analysis of splice variants generated by the ENCODE consortium [30] revealed little evidence that alternative splice variants commonly give rise to functional isoforms. In the case of frameshifts, if both alternative open reading frames lead to functional proteins, one would expect the polymorphism or divergence level in all three codon positions to be the same [30]. Few splice variants in the ENCODE analysis displayed this property [30]. Our data are consistent with previous results. When looking at all 42 organisms in our analysis summed together (for a total of 7,115 CEs), CEs are more likely to have lengths that are a multiple of three (45% in all species examined), but over half of all CEs have lengths that leave remainders of one (28%) or two (27%) when divided by three.

Less has been reported about the functional impact of RIs. In humans, many RIs have been shown to be not merely partially spliced transcripts or splicing errors [31]. They were shown to have evidence of coding potential (having higher GC content than other introns, having codon usage more like exons, and having a lower frequency of stop codons). Many human RIs participate in coding for a protein domain (a smaller fraction than for exons, but a greater fraction than for constitutive introns) [31]. However, not all RIs in higher eukaryotes are necessarily functional. In plants, many RIs were shown to introduce premature termination codons or frameshifts [12].

The prevalence of RIs in all organisms we analyzed provides an opportunity to assess the possibility of a functional role for these observed events. Our analysis reveals that RIs do not display a preference for preserving reading frames: the lengths of all 11,925 observed RIs were roughly equally distributed between intron lengths evenly divisible by three (34%), and intron lengths with remainders of one (34%) and two (33%) when divided by three. Among the ten organisms with greater than 500 RIs, the number evenly divisible by three is 34 ± 2%, with a slightly higher value of 37% for *D. rerio*.

Though our analysis shows little evidence of frame preservation in RIs, we do see weak selection for coding potential. Between the closely related species of *C. neoformans *and *Coccidioides immitis *dN/dS ratios for concatenated RIs showed weak but significant evidence of conservation at the amino acid level (*p *< 0.001 for *C. neoformans *and p = 0.05 for *C. immitis*; Table 5). We also observe significantly fewer in-frame stop codons within RIs than in constitutive introns, with 23% fewer (*p *< 0.0001) in *C. immitis*, and 20% fewer (*p *= 0.02) in *C. neoformans *(Table 5). We observe no significant functional group over-representation (Table S3 in Additional data file 4).

**Table 5:** dN/dS ratio and stop codon density for Ris

	dN/dS ratio for RIs	dN/dS for constitutive introns	*p *value	Stop codon density for RIs	Stop codon density for constitutive introns	*p *value
*C. neoformans*	0.8588	1.0008	<0.001	0.0124	0.01534	0.02
*C. immitis*	0.7743	0.9083	0.049	0.01153	0.01507	<0.0001

We thus see some evidence for coding potential in RIs, but taken together with previous observations of CEs, our results suggest that the majority of observed splice variants are unlikely to give rise to functional proteins. It has been proposed that splice variants leading to frameshifts or truncated proteins may be due in part to artifacts associated with EST library construction or sequencing. However, the universality of such disrupting variants across the many independent data sets and kingdoms analyzed here - and the occurrence of such disruptions associated with both RIs and CEs - suggest that these events occur naturally and frequently. In humans, plants, and fungi, transcripts containing premature stop codons are targeted for degradation through the process of nonsense mediated decay [32,33]. The widespread occurrence of premature stop codons in human splice variants has led to the hypothesis that unproductive splicing and translation may be pervasive [34]. Our results are consistent with this hypothesis.

### Retained introns are associated with weak splice sites

Studies in mammals have demonstrated that splice sites adjacent to CEs and RIs are associated with weak splice site signals [28,35-40]. We evaluated RI information content in plants, fungi and protists and report results in agreement with previous studies.

We quantified 3' and 5' splice site strength by calculating the information content of the splice sites (see Materials and methods; Additional data file 2; Table S1 in Additional data file 4). We found significantly lower information content on either side of retained introns than constitutive introns for all 40 organisms in our analysis (*P *values 3.2e-9 and 2.1e-11, respectively, calculated from *t*-test). Overall, RI 5' splice sites had 1.9 ± 1.4 bits (24%) less information content, while their 3' splice sites had 0.9 ± 0.8 bits (17%) less (see data in Table S1 in Additional data file 4 and sequence logos [41] in Additional data file 2.) We observed the largest differences between RIs and constitutive introns in animals. The average differences in information content for 5' and 3' splice sites were, respectively, 2.3 and 1.0 bits for animals, 2.1 and 0.9 bits for fungi, 1.6 and 0.8 bits for protists, and 1.4 and 0.6 bits for plants.

While weak splice sites, such as those we observe in our RIs, have been associated with functional RIs and CEs [28,35-40], they are also expected to lead to greater occurrence of incomplete splicing [18]. Therefore, weaker splice sites are uninformative as to whether these RIs are functional or merely incomplete splicing.

## Discussion

### Splice variation reveals mechanisms of splice site recognition

The variation we observe in the CE fraction, the correspondence of this variation with average intron length, and the size constraints observed in both RIs and CEs can be parsimoniously explained by proposing differences in the proportion of splice junctions each organism recognizes via ED and ID. Organisms with a high CE fraction presumably splice predominantly via ED, whereas organisms with a low fraction presumably have a preference for ID-mediated splicing. Our results suggest that the fraction of introns recognized by ID and ED vary extensively across kingdoms, yet both mechanisms play a role in splicing in all phylogenetic groups.

In animals, exons are short relative to introns (Table 4) and CEs are more common than RIs (Table 3). This pattern is consistent with the hypothesis that splice junctions in animals are primarily recognized through ED, as has been previously demonstrated in vertebrates [19]. Moreover, the predominance of ED in these species predicts that CEs should be approximately as long as constitutive exons, because they are spliced the same way and, thus, are subject to the same length constraints. This is precisely the behavior we observe (Figure 4).

In fungi and protists, conversely, introns are short relative to exons (Table 4) and RIs far outnumber CEs. We propose that these groups primarily recognize splice junctions using ID (Figure 5). Because the splicing machinery in these organisms recognizes RIs in the same way it recognizes constitutive introns, we expect both types of intron should be subject to the same length constraints. Supporting this hypothesis, Figure 5 shows that constitutive introns in these species are similar in length to RIs. Intron definition has been demonstrated experimentally in the yeasts *Saccharomyces *and *Schizosaccharomyces *[17,27], and in plants [42]. Our analysis extends this result to all ascomycetes, as well as basidiomycetes and zygomycetes, and suggests that ID is not simply a characteristic of the derived yeasts. Furthermore, due to their high prevalence of RIs, we predict that ID predominates in the basidiomycete *C. neoformans*, which possesses 5.4 introns per gene on average, as well as the protozoan *Tetrahymena thermophila*, which has 3.3 introns per gene. ID is not associated with low intron density *per se*.

In plants, intron lengths vary widely, and individual species show substantial numbers of introns both greater and less than 200 bp in length (Table 4). Correspondingly, we observe that RIs and CEs both occur in sizeable quantities in this group. We thus propose that ID and ED both play significant roles in splice site recognition in plants.

While ED is most common in animals and ID dominates in fungi and protists, nearly all species analyzed show evidence for using both mechanisms. ID and ED have previously been shown to operate within the same species and indeed within the same gene [21]. We thus propose that the intron and exon length distributions in any organism are each sums of two distributions: one made up of shorter introns recognized by ID and the longer exons that surround them, and one made up of shorter exons recognized by ED and the surrounding longer introns. When we examine the lengths of CEs, we sample from only one of these two distributions: the subset of exons recognized by ED. When we examine the lengths of RIs, we sample from the length distribution of introns recognized by ID. Both these distributions are biased to be short, and this length bias is particularly noticeable in organisms where these splice variants are rare. Finally, when we look at the lengths of introns surrounding CEs, we are primarily sampling from the distribution of introns associated with ED. This last distribution tends to be long (Additional data file 3), as is the length distribution of exons surrounding RIs, which are associated with ID. (See Additional data file 3 for more detail on intron and exon length distributions in CEs and RIs.)

Importantly, our model of varying levels of ID or ED splice-site recognition explains the variation we see in CE fraction, whether or not individual variants lead to functional messages. As shown in Figure 1a, ID mis-recognition of a single splice site should lead to intron retention, as has been demonstrated by splice site mutation experiments in *Drosophila *and *Schizosaccharomyces *[17,18]. Creating a CE via ID, however, would theoretically require coordinated mis-recognition of two splice sites and pairing of splice sites over a greater distance. If this distance were greater than 200 bp, pairing with ID would be considerably hindered [24]. Similarly, as experimentally demonstrated and shown in Figure 1b, ED mis-recognition of a single splice site leads to a CE [19]. The hypothetical generation of RIs under ED would require multiple mis-recognitions and pairing of splice sites belonging to two possibly distant exons.

Thus, if many non-functional splice variants arise as a consequence of incorrect or incomplete splice site recognition, we would nonetheless expect that splice sites recognized by ID would more commonly give rise to RIs while those recognized by ED would more commonly give rise to CEs. Non-functional splice variants, therefore, are as informative as functional ones when considering the question of how splice sites are recognized. This is fortunate, as it is unclear at present what proportion of observed splice variation is indeed functionally significant. Splice variants and their characteristics, then, provide insight into the underlying mechanisms of splice site recognition, irrespective of whether such variants are functional or biological errors.

### Evolutionary implications of splice variation

The varying prevalence of CEs and RIs across major eukaryotic groups raises evolutionary questions about modern variation in splice recognition among lineages, as well as the nature of splice site recognition in the last common eukaryotic ancestor. As RIs and CEs are exhibited in almost every organism we studied, it is likely that almost all extant eukaryotes are capable of recognizing splice sites via both ID and ED. The last common eukaryotic ancestor, then, may also have been capable of both types of splice site recognition. Indeed, Collins and Penny [43] report that most of the key components of the spliceosome were present in the last common eukaryotic ancestor. If the minimal mechanistic requirements to support both ID and ED were present in the last common eukaryotic ancestor, which was more prevalent: ID or ED? In our analysis, we note that intron density has less effect on the types of observed splice variation than does intron length. For example, the fungus *C. neoformans *recognizes splice sites almost exclusively by ID, despite having more introns per gene than many plants where ED is prevalent. Thus, whether the last common eukaryotic ancestor recognized splice sites predominantly via ID or ED is probably not a question of how many introns it had but rather how long its introns were. As very long introns appear to be a derived feature associated with multicellularity, we may speculate that the last eukaryotic common ancestor had introns similar in size to most protists, and, therefore, probably employed ID more than ED.

Evidence pertaining to the evolutionary origin of introns also suggests a prevalence of ID in early eukaryotes. Similarities in the splicing mechanism between self-splicing group II introns in prokaryotes and spliceosomal eukaryotic introns suggest that the former may have begat the latter [44-48]. As group II introns are mobile genetic elements that spread through retrotransposition, the ancestors of spliceosomal introns were probably self-contained in terms of their signals for excision, and would not likely have relied on splicing factors embedded in flanking coding sequence (as may be required for ED [49]). Therefore, the earliest spliceosomal introns may have employed a method of splice site recognition most similar to ID.

Recent molecular evidence suggests that SR (serine-arginine-rich) proteins may be associated with the ascendance of ED in animals and plants [50]. SR proteins bind to RNA sequences and assist in spliceosome assembly for both constitutively and alternatively spliced introns. They have been shown to enhance the splicing efficiency of introns with suboptimal splice signals in *S. pombe *[51]. Additionally, Shen and Green [52] have recently shown that SR proteins can rescue splicing of introns with suboptimal splice signals if those proteins are directed to bind to exonic mRNA sequence in *S. cerevisiae*, a species that has lost all native SR proteins. If the experimental binding of SR proteins to pre-mRNA sequences is indicative of the binding of SR proteins to exonic splicing enhancers in organisms where ED is prevalent, the fundamental splicing machinery of ID and ED may be closely related. Along these lines, Ram and Ast [50] speculate that the primary role of SR proteins has changed over time. In early eukaryotes, SR proteins assist in the recognition of suboptimal ID introns, while in higher eukaryotes, they bind to exonic splicing enhancers. This role change shifts the placement of the basal splicing machinery across exons instead of across introns and enables ED using the same spliceosomal machinery employed for ID [50].

The SR protein family has expanded in multicellular eukaryotic lineages [53], and this proliferation may have facilitated the widespread conversion of introns in those lineages from ID to ED. However, it is unclear what underlying neutral or selective forces could be responsible for this shift. Changes in genome size may have played a role. Multicellular eukaryotes generally exhibit larger genomes than unicellular eukaryotes, and organisms with large genomes tend to have long introns [54,55]. Because ID is only effective for introns less than approximately 250 bp [24], an upward trend in genome size in multicellular lineages (resulting from a reduced deletion rate, increased transposon activity, or both) could have favored ED introns that were spliceable in the face of this mutational pressure.

Regardless of which evolutionary forces are responsible for their ascendance, CEs produced through exon definition in multicellular eukaryotes allow for much greater flexibility and combinatorial complexity of alternatively spliced transcripts than would RIs recognized by ID. For example, the large number of transcripts (>30,000) that can be produced by the *Dscam *gene in *D. melanogaster *is facilitated by independent splicing of CEs [56]. The abundance of CEs enabled by ED in the human proteome may help to explain why the human genome contains only about as many genes as that of the worm or fly, despite the (admittedly biased) perception of our own much greater organismal complexity [57].

## Conclusion

Using EST and cDNA data from 42 organisms, we find the prevalence of RIs and CEs to vary significantly across major eukaryotic groups, strongly suggesting that the underlying mode of splice site recognition (ID versus ED) also varies in prevalence across eukaryotes. Our results show that RIs, which are present in every organism we analyzed, are more widespread than previously thought. We also find a strong relationship between intron length and the prevalence of splice variation: the fraction of introns greater than 200 bp is correlated with the CE fraction. Shorter introns (<200 bp), such as those found in fungi and protists, are more likely to be recognized by ID. In all 23 fungi and protists that we examined, we observed that RIs are more common than CEs. In contrast, shorter exons surrounded by longer introns (>200-250 bp), such as those found in animals, are more likely to be recognized by ED. In the 13 multicellular animals in our analysis, CEs occur much more frequently, sometimes in greater numbers than RIs. The six plants in our analysis exhibited intermediate intron lengths, having more CEs than fungi and more RIs than animals.

We conclude that ID and ED are likely both present to some degree in all eukaryotes. We conclude that splicing proceeds primarily by ID in fungi and unicellular protists, due to the overwhelming majority of RIs and paucity of CEs observed in these organisms, as well as their short intron lengths and longer exon lengths. In contrast, splice sites in multicellular animals are recognized primarily via ED, due to the larger numbers of CEs observed, as well as these species' longer introns and shorter exons. However, the molecular mechanisms underlying the two different forms of recognition (ED and ID) are still unclear.

These findings help to reveal the complex interplay of selective constraints and mutational pressures underlying eukaryotic genome architecture, and improve our understanding of why eukaryotic genomes exhibit so much variation. Further sequencing of additional organisms, especially those that exhibit both unicellular and multicellular properties, will help to disentangle the effects of multicellularity, genome size, and intron length on the mechanisms of splice site recognition.

## Materials and methods

### EST alignments

All EST and assembly data were publicly available and downloaded from the Broad Institute [58], GenBank, J Craig Venter Institute/The Institute for Genomic Research [59], Joint Genome Institute [60] or VectorBase [61] (Tables 1 and 2). We used BLAT [62] version 33 to align the ESTs to genomic sequence using the following parameters: --minIdentity = 95 --minScore = 50 --queryType = rna. We set the --maxIntron parameter to the longest annotated intron in each species.

We filtered the resulting alignments using the following criteria: each alignment must contain at least one canonical splice site (GT:AG, CG:AG, AT:AC); must have no non-canonical splice sites; must have ≥ 95% nucleotide identity; must not have more than nine consecutive insertions; and must not have more than nine consecutive deletions outside of an intron. We also required three or more exact matches at every intron-exon boundary [12]. If a single EST aligned to more than one location on the genome, we only considered the alignment with the highest score. To guard against redundant input data, if multiple alignments in the same locus had the same sequence after trimming, we disregarded all but one of them.

We discarded all unspliced alignments to prevent labeling pre-spliced transcripts as splice variants. Unspliced ESTs show no evidence of having been processed by the spliceosome, and may represent pre-spliced transcript fragments. It is difficult to distinguish between an unspliced EST that has been processed and an unspliced EST that has not, and thus we cannot report how many processed ESTs we discarded. However, if most unspliced ESTs have already been processed by the spliceosome, we would expect to find more of them in organisms with very few introns and/or very long exons. We found no such correlation in either case (Additional data file 5). We conclude that a substantial fraction of the unspliced ESTs represent pre-spliced ESTs, and, therefore, that unspliced ESTs are not a reliable indicator of splice variation.

The number of RIs changed substantially depending on whether we included or excluded unspliced ESTs, while the numbers of CEs and competing 3' and 5' splice sites changed very little. We believe that previous reports that do not exclude unspliced ESTs overestimate the frequency of intron retention.

After aligning the ESTs and filtering them, we built transcripts and transcript fragments using CallReferenceGenes, an unpublished tool used in the Broad Institute's genome annotation pipeline since 2005. Several previous papers provide an in-depth discussion of the problem [12,63]. We briefly sketch our algorithm here. The source code for CallReferenceGenes, as well as the code that labels alternative splice forms, is included in Additional data file 6.

First, we partition the alignments into clusters, so that every alignment has exon-exon overlap with at least one other alignment in the cluster, and no alignment has exon-exon overlap with any alignment outside its cluster. Alignments that overlap no other alignments are ignored.

Next, for each cluster, we compare every alignment to every other alignment that overlaps it. (Here, as opposed to the previous step, we also consider exon-intron overlaps.) Relationships are directional, and are given one of three labels: 'conflicts', 'extended-by', or 'includes'. Two alignments conflict if any base in the region of overlap is exonic in one alignment and intronic in the other. If they do not conflict, one alignment extends another if it ends after the other and does not begin before it. Lastly, one alignment includes another if it does not conflict with it, starts before it, and ends after it.

At this stage the cluster is represented by a graph of nodes (representing alignments) and labeled edges (representing their relationships). To turn this into a more tree-like representation that is easier to traverse, we build an ordered list of alignments. We sort them by 3' coordinate, in ascending order. In the case where we have two alignments with identical 3' coordinates, we sort by 5' coordinate, again in ascending order. In this way, no alignment is extended by any element that sorts before it. An alignment usually, though not always, sorts after the alignments it includes.

To improve performance, we prune redundant relationships from the graph. We apply a series of heuristics to reduce the number of paths from an alignment to its descendants. These 'trimming rules' include: first, if A is extended by B, and C extends both A and B, and there is no element D such that B conflicts with D and C does not, then any path containing A and B will also contain C. The extended-by relationship from A to C is therefore redundant. Second, if A is included by B, and there is no element D such that B conflicts with D and A does not, then any path containing A will also include B. All extended-by relationships terminating in A are redundant. Third, if A is extended by B and both are included by C, and every element D that conflicts with C also conflicts with either A or B, then any path containing both A and B will also contain C. Thus, the extended-by relationship from A to B is redundant.

We traverse the list bottom-up so that every element is pruned before any element it extends. There will be multiple paths from a parent to a given child if a splice variation lies between them. If no splice variation occurs between them, generally, there will be one path, but the above rules are not exhaustive and some duplicate paths may remain after pruning. As we traverse the list and prune it, we track, per alignment, all alignments that can be reached through it (for example, all its extenders and includees, as well as their extenders and includees, and so on). We call these sets of alignments the 'descendants' of each alignment. (Note that pruning never reduces the membership of these sets, only the number of edges between them.)

After ordering and pruning, we can traverse the list of alignments left-to-right, following 'extended-by' and 'includes' links to build paths linking splice-compatible alignments. To do this, we do the following. First, find starts - a start is any element that extends no other element and has at least one descendant that cannot be reached through any previously discovered start. Second, walk the tree - treating each start, in turn, as a root of a subtree, traverse extension and inclusion links as edges in a graph; each unique path represents (a fragment of) a transcript. Third, remove sub-paths - if all the alignments in one path are present in another, we discard the one containing fewer alignments. Fourth, overlapping paths with distinct alignments represent splice variants of the same gene.

Given two overlapping transcripts A and B, all bases in the region of overlap will be in one of four states: I, A and B are exonic; II, A is exonic; III, B is exonic; IV, neither is exonic. We group all adjacent states into a column with a single label, then use a sliding-window approach, across three such columns, to compare overlapping transcripts. CEs, RIs, and competing 5' and 3' splice sites all have a different signature appearance. CEs appear as IV:II:IV and IV:III:IV; RIs appear as I:II:I and I:III:I; competing 5' and 3' splice sites appear as I:II:IV, I:III:IV, IV:II:I, and IV:III:I.

We filter the set of splice variants by requiring that every base in the variant region be exonic in at least one alignment and intronic in at least one alignment. Because EST data are fragmentary we cannot be sure that initial or terminal exons are complete. To ensure accurate labeling as well as reliable length statistics, we require that any exon in the alternatively spliced region not be an initial or terminal exon. Finally, we require that every alignment spanning the region conform to either the major or minor variant. The reported length of each splice variant is simply the width of the center column in the windows described above.

We also generated a list of constitutive introns and exons as a control. These are introns and exons predicted from loci where no ESTs conflict. Furthermore, every base within these constitutive elements must have ten or more ESTs supporting it. Note that there is no way to tell for sure that any exon or intron never exhibits splice variation; this method simply identifies loci where splice variation has never been seen, and if present, is presumably rare.

### Testing retained introns for evidence of selection at the codon level

We used a comparative approach to test retained introns for purifying selection at the codon level using two groups of fungi, where genome sequences at suitable evolutionary distances were available. We examined all four sequenced serotypes of *C. neoformans *(JEC21, H99, R265 and WM276) in one group, while the second group consisted of *C. immitis *and the C735 strain of *Coccidioides posadasii*. Orthology of genes was determined using a reciprocal-best-BLAST criterion, while the alignment of orthologs was performed using ClustalW [64]. Alignments of retained orthologous introns were concatenated to enhance power for detecting selection. Prior to concatenation, splice donor and acceptor sites were removed, and the 5' and 3' ends of each intron alignment were padded with gaps in order to preserve the native reading frames of introns. We used a 100 bp cutoff for retained introns in *C. neoformans *and 150 bp in *C. immitis*. Since 95% of the introns we identified in each organism were less than these cutoffs, we used a cutoff to eliminate unusually long introns. We analyzed 477 and 389 orthologous intron alignments in *C. neoformans *and *C. immitis*, respectively. The dN/dS ratio of concatenated intron alignments was calculated using the codeml program (model M0) in the PAML 3.15 software package [65]. The probability that the resulting dN/dS ratios were significantly less than one, indicating purifying selection at the codon level, was calculated using a bootstrapping approach with a set of control introns. We identified 672 and 662 control introns in *C. neoformans *and *C. immitis*, respectively. We randomly resampled, with replacement, from the control introns to create 1,000 concatenated control alignments approximately equal in length to the concatenated retained intron alignments in each taxonomic group. Then, we used codeml to calculate the dN/dS ratio exhibited by each resampled control alignment to determine the probability of observing dN/dS ratios as low as or lower than those exhibited by the retained introns in each taxonomic group, under a null hypothesis that they are non-coding.

## Abbreviations

CE, cassette exon; ED, exon definition; EST, expressed sequence tag; ID, intron definition; RI, retained intron; SR, serine-arginine-rich.

## Authors' contributions

AMM and MDP contributed equally. MDP wrote the EST alignment software and contributed to writing the paper. AMM performed the analysis and drafted and finalized the manuscript. DEN performed the dN/dS and stop codon density analyses, and contributed to writing the paper. JEG initiated and supervised the study, and revised the manuscript.

## Additional data files

The following additional data are available with the online version of this paper. Additional data file 1 is a figure showing the frequencies of different forms of splice variation. Additional file 2 is a figure showing sequence logos for splice sites for both RIs and controls. Additional data file 3 shows intron and exon length distributions for six example organisms. Additional data file 4 contains supplementary tables detailing the differences in information content between RIs and controls (Table S1), intron and exon lengths from annotations (Table S2), and details of our analysis of functional group enrichment of RIs (Table S3). Additional data file 5 is a spreadsheet containing a comparison of alternative splicing events for the situations where unspliced ESTs are included as well as discarded. This spreadsheet also includes data for a higher-confidence dataset requiring a greater number of ESTs supporting each predicted alternative splicing event. Additional data file 6 contains computer source codes for the CallReferenceGenes program, as well as the code that labels alternative splice forms. These codes can be inspected but will not be functional without the rest of the Broad Institute's Calhoun environment.

## Supplementary Material

Additional data file 1 The data for *H. sapiens *were taken from a previous study [8]. Click here for file

Additional data file 2 **(a) **Sequence logos [41] for 3' splice sites of RIs and control introns. **(b) **Sequence logos [41] for 5' splice sites of RIs and control introns. For 5' splice sites, we show sequence logos for six intronic and three exonic bases. For 3' splice sites, we show sequence logos for three intronic and one exonic bases. Click here for file

Additional data file 3 Six example organisms with large numbers of splice variants were chosen and their normalized intron and exon length distributions are plotted here. **(a) **CE distributions are shifted towards shorter lengths than the control exon length distribution, because of constraints on exon length imposed by ED. **(b) **The peak at short intron lengths is very similar in RIs and constitutive introns, because this short intron peak is primarily made up of introns recognized by ID. In contrast, almost no introns surrounding CEs have lengths close to this 'intron-definition peak' - almost all of them are spread out over a wide range of longer intron lengths, and have low values in the area of the short intron-length ID peak shown here (0-200 bp). Click here for file

Additional data file 4 Table S1: differences in information content between RIs and controls. Table S2: intron and exon lengths from annotations. Table S3: details of our analysis of functional group enrichment of RIs. Click here for file

Additional data file 5 A comparison of alternative splicing events for the situations where unspliced ESTs are included as well as discarded. We also include data for a higher-confidence dataset requiring a greater number of ESTs supporting each predicted alternative splicing event. Click here for file

Additional data file 6 We include the source code for the CallReferenceGenes program, as well as the code which labels alternative splice forms. These codes can be inspected but will not be functional without the rest of the Broad Institute's Calhoun environment. These are included as Unix tar files. Click here for file
