# Supplementary figures and images for: Cross-kingdom patterns of alternative splicing and splice recognition
Source: Genome Biol. 2008 Mar 5;9(3):R50. doi: 10.1186/gb-2008-9-3-r50 (PMC2397502; doi:10.1186/gb-2008-9-3-r50)

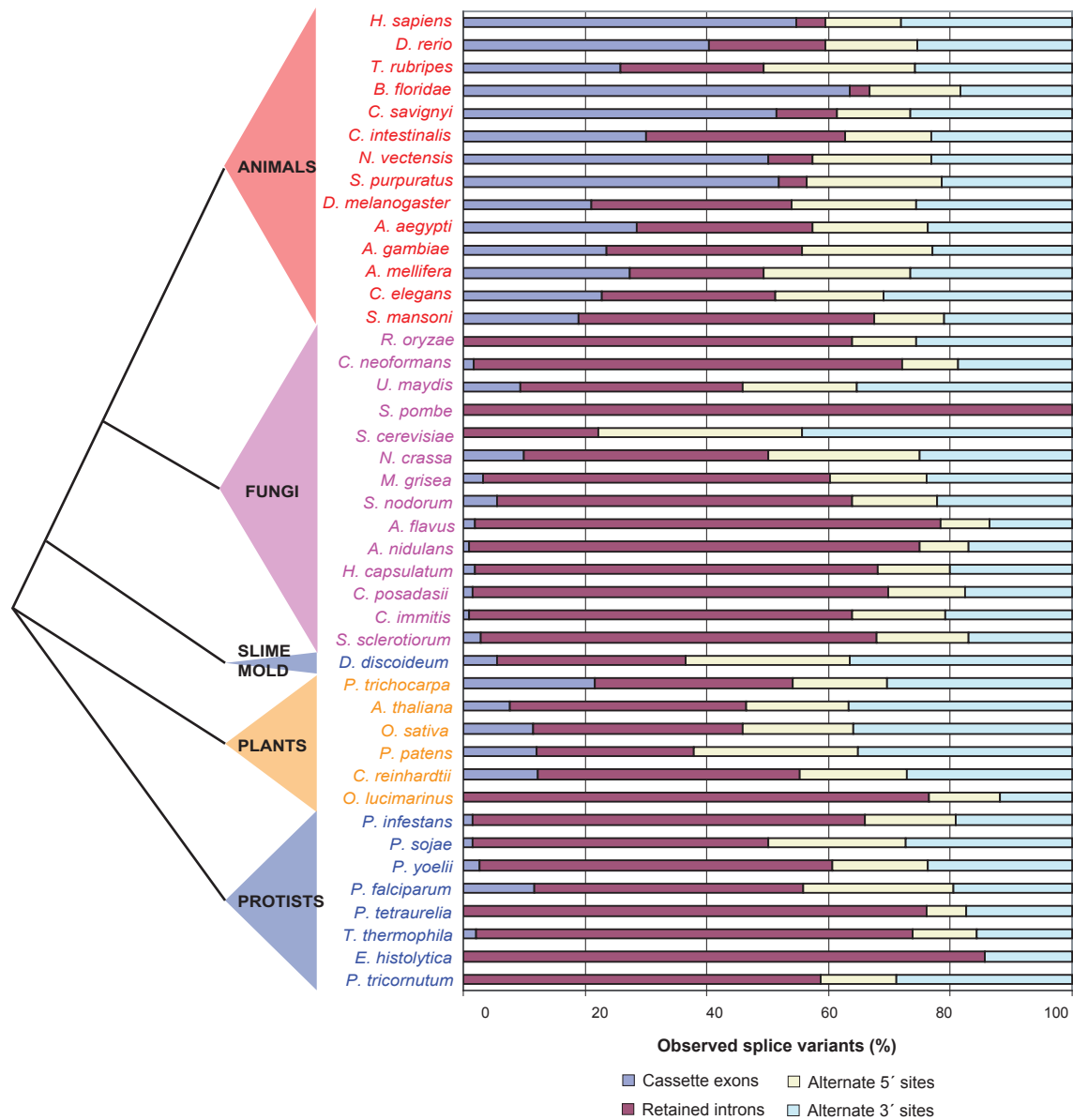

Supplement: Additional data file 1 — The data for H. sapiens were taken from a previous study [8]. [file gb-2008-9-3-r50-S1.pdf]

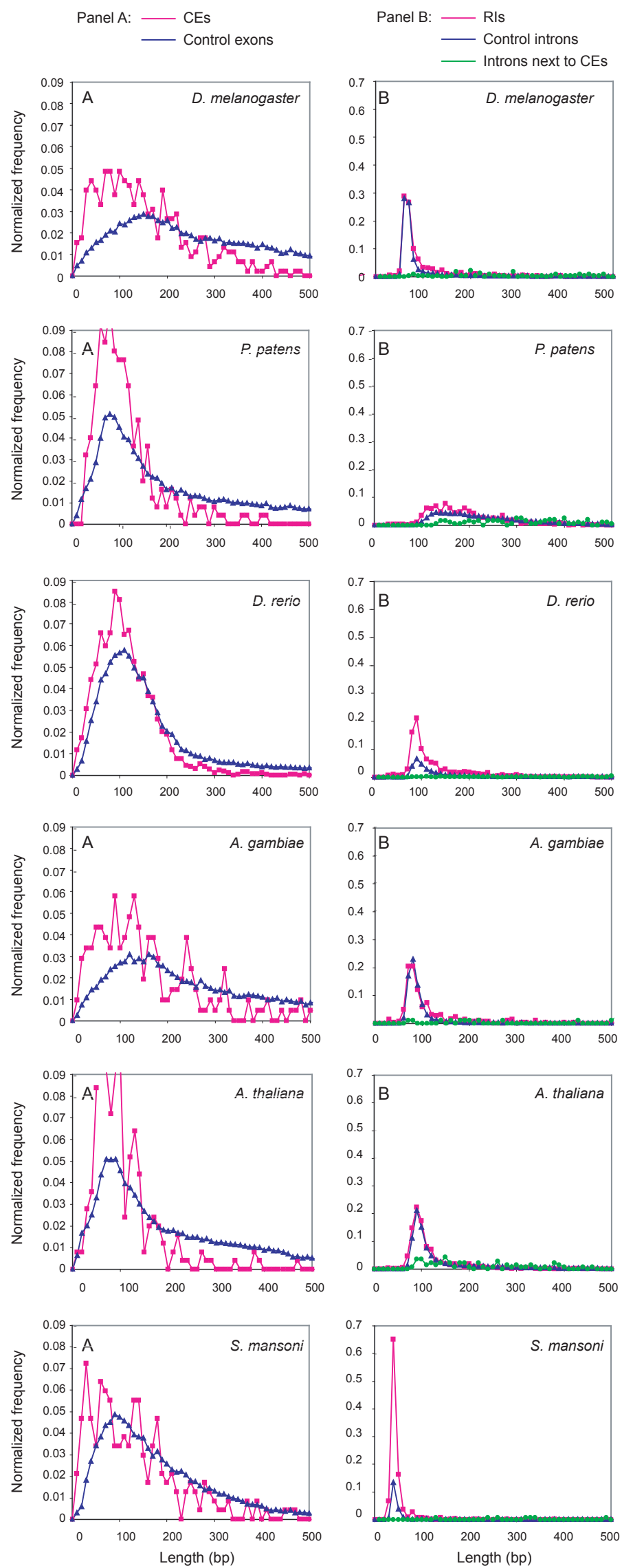

Supplement: Additional data file 3 — Six example organisms with large numbers of splice variants were chosen and their normalized intron and exon length distributions are plotted here. (a) CE distributions are shifted towards shorter lengths than the control exon length distribution, because of constraints on exon length imposed by ED. (b) The peak at short intron lengths is very similar in RIs and constitutive introns, because this short intron peak is primarily made up of introns recognized by ID. In contrast, almost no introns surrounding CEs have lengths close to this 'intron-definition peak' - almost all of them are spread out over a wide range of longer intron lengths, and have low values in the area of the short intron-length ID peak shown here (0-200 bp). [file gb-2008-9-3-r50-S3.pdf]
