# Supplementary material for: Cross-kingdom patterns of alternative splicing and splice recognition
Source: Genome Biol. 2008 Mar 5;9(3):R50. doi: 10.1186/gb-2008-9-3-r50 (PMC2397502; doi:10.1186/gb-2008-9-3-r50)

McGuire et al.
Supplemental Figure 2

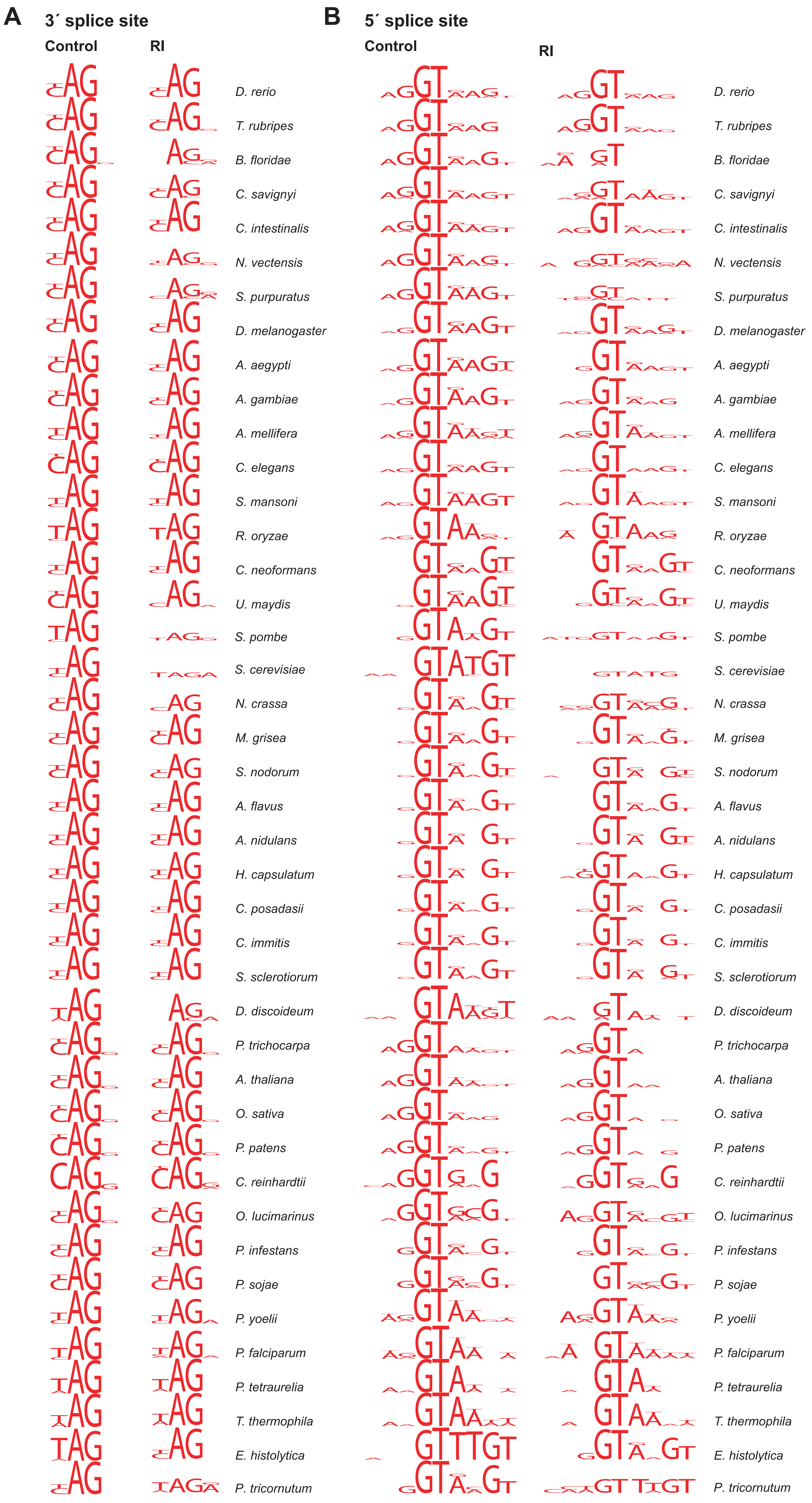

Supplement: Additional data file 2 — (a) Sequence logos [41] for 3' splice sites of RIs and control introns. (b) Sequence logos [41] for 5' splice sites of RIs and control introns. For 5' splice sites, we show sequence logos for six intronic and three exonic bases. For 3' splice sites, we show sequence logos for three intronic and one exonic bases. [file gb-2008-9-3-r50-S2.pdf]
