# Supplementary material for: Cross-kingdom patterns of alternative splicing and splice recognition
Source: Genome Biol. 2008 Mar 5;9(3):R50. doi: 10.1186/gb-2008-9-3-r50 (PMC2397502; doi:10.1186/gb-2008-9-3-r50)
Supplement: Additional data file 4 — Table S1: differences in information content between RIs and controls. Table S2: intron and exon lengths from annotations. Table S3: details of our analysis of functional group enrichment of RIs. [file gb-2008-9-3-r50-S4.doc]

**Supplementary Table 1 - Difference in information content between RIs and controls**

|  | RI (3’) | Controls (3’) | RI (5’) | Controls (5’) |
| --- | --- | --- | --- | --- |
| *D. rerio* | 4.61 | 4.98 | 6.06 | 7.41 |
| *T. rubripes* | 4.50 | 5.18 | 6.15 | 7.42 |
| *B. floridae* | 2.98 | 5.24 | 3.97 | 7.85 |
| *C. savignyi* | 3.49 | 5.20 | 5.26 | 7.55 |
| *C. intestinalis* | 4.73 | 5.03 | 6.48 | 7.66 |
| *N. vectensis* | 2.62 | 4.92 | 4.79 | 7.86 |
| *S. purpuratus* | 3.15 | 5.06 | 3.38 | 8.48 |
| *D. melanogaster* | 4.49 | 4.86 | 6.13 | 8.02 |
| *A. aegypti* | 4.38 | 5.03 | 6.17 | 8.38 |
| *A. gambiae* | 4.25 | 4.97 | 5.70 | 8.31 |
| *A. mellifera* | 4.22 | 4.89 | 6.53 | 7.78 |
| *C. elegans* | 4.90 | 5.38 | 5.60 | 7.21 |
| *S. mansoni* | 4.50 | 4.67 | 6.30 | 8.20 |
| *R. oryzae* | 4.36 | 5.04 | 6.28 | 7.92 |
| *C. neoformans* | 4.42 | 4.63 | 6.83 | 7.91 |
| *U. maydis* | 3.97 | 5.01 | 6.11 | 9.18 |
| *S. pombe* | 1.92 | 4.93 | 3.82 | 8.73 |
| *S. cerevisiae* | 1.80 | 4.38 | 3.01 | 10.18 |
| *N. crassa* | 2.93 | 4.81 | 5.32 | 7.86 |
| *M. grisea* | 4.57 | 4.72 | 6.30 | 7.60 |
| *S. nodorum* | 3.48 | 4.81 | 5.25 | 7.61 |
| *A. flavus* | 4.37 | 4.65 | 6.39 | 7.39 |
| *A. nidulans* | 4.40 | 4.69 | 6.65 | 7.19 |
| *H. capsulatum* | 4.06 | 4.66 | 6.46 | 7.06 |
| *C. posadasii* | 4.41 | 4.62 | 5.99 | 6.89 |
| *C. immitis* | 4.37 | 4.65 | 5.85 | 6.96 |
| *S. sclerotiorum* | 4.47 | 4.61 | 6.46 | 7.46 |
| *D. discoideum* | 3.39 | 4.97 | 5.21 | 8.95 |
| *P. trichocarpa* | 4.76 | 5.30 | 5.79 | 7.02 |
| *A. thaliana* | 4.70 | 5.11 | 5.88 | 6.99 |
| *O. sativa* | 4.90 | 5.32 | 5.64 | 6.84 |
| *P. patens* | 5.08 | 5.62 | 5.74 | 6.98 |
| *C. reinhardtii* | 5.72 | 6.19 | 7.53 | 8.92 |
| *O. lucimarinus* | 3.78 | 4.95 | 6.57 | 8.67 |
| *P. infestans* | 4.47 | 4.78 | 6.19 | 7.13 |
| *P. sojae* | 4.16 | 4.99 | 6.38 | 7.59 |
| *P. yoelii* | 4.10 | 4.91 | 6.87 | 7.80 |
| *P. falciparum* | 4.23 | 4.96 | 7.17 | 7.82 |
| *P. tetraurelia* | 4.86 | 5.09 | 6.85 | 7.59 |
| *T. thermophila* | 4.71 | 4.88 | 7.62 | 8.63 |
| *E. histolytica* | 3.36 | 5.29 | 7.00 | 11.00 |
| *P. tricornutum* | 4.40 | 4.65 | 8.06 | 9.05 |
| average | 4.12 | 4.97 | 5.99 | 7.93 |

**Supplementary Table S2 - Intron and exon lengths from annotations.**

|  | Avg intron length & std. dev^*^ | | Frac introns > 200 bp^**^ | Avg Exon length & std. dev^§^ | | Avg internal exon length^†^ | Annotations used |
| --- | --- | --- | --- | --- | --- | --- | --- |
| *A. aegypti* | 4643 | ± 10121 | 47% | 343 | ± 454 | 348 | ENSEMBL release 45 [1, 2] |
| *A. flavus* | 122 | ± 149 | 13% | 439 | ± 614 | 336 | Broad Institute [3] (Matt Pearson, personal communication) |
| *A. gambiae* | 1162 | ± 4358 | 32% | 345 | ± 458 | 336 | ENSEMBL release 45 [1, 2] |
| *A. mellifera* | 1235 | ± 8475 | 37% | 253 | ± 343 | 244 | None used |
| *A. nidulans* | 102 | ± 100 | 11% | 418 | ± 606 | 343 | Genbank AACD01000001:AACD01000248 |
| *A. thaliana* | 159 | ± 163 | 19% | 215 | ± 279 | 159 | Genbank NC_003070:NC_003076 |
| *B. floridae* | 1259 | ± 2688 | 91% | 207 | ± 299 | 162 | JGI [4] |
| *C. elegans* | 323 | ± 738 | 34% | 205 | ± 246 | 219 | Genbank NC_003279:NC_003284 |
| *C. immitis* | 139 | ± 124 | 22% | 358 | ± 547 | 302 | Broad Institute [3] |
| *C. intestinalis* | 547 | ± 1121 | 77% | 150 | ± 149 | 141 | ENSEMBL release 45 [1, 2] |
| *C. neoformans* | 68 | ± 55 | 2% | 252 | ± 320 | 232 | Genbank |
| *C. posadasii* | 82 | ± 55 | 3% | 496 | ± 667 | 380 | TIGR/JCVI [5] |
| *C. reinhardtii* | 370 | ± 532 | 63% | 192 | ± 248 | 155 | JGI [4] |
| *C. savignyi* | 685 | ± 1006 | 81% | 143 | ± 122 | 138 | ENSEMBL release 45 [1, 2] |
| *D. discoideum* | 142 | ± 154 | 13% | 636 | ± 983 | 426 | Genbank NC_001889, NC_007087:NC_007092 |
| *D. melanogaster* | 1022 | ± 4126 | 34% | 366 | ± 515 | 350 | Genbank NC_004353, NC_004354, NC_037436, NC_033777:NC_0033779 |
| *D. rerio* | 2768 | ± 10,884 | 69% | 171 | ± 267 | 151 | Genbank NC_007112:NC_007136 |
| *E. histolytica* |  |  |  |  |  |  | None used |
| *H. capsulatum* | 142 | ± 111 | 21% | 372 | ± 519 | 324 | Broad Institute [3] |
| *H. sapiens* | 5424 | ± 17,616 | 87% | 164 | ± 257 | 144 | Genbank |
| *M. grisea* | 139 | ± 118 | 17% | 494 | ± 689 | 390 | Broad Institute [3] |
| *M. musculus* | 4554 | ± 16,502 | 86% | 170 | ± 261 | 146 | Genbank |
| *N. crassa* | 121 | ± 101 | 15% | 511 | ± 715 | 449 | Genbank NW_047255 |
| *N. vectensis* | 801 | ± 1495 | 79% | 212 | ± 283 | 140 | JGI [4] |
| *O. lucimarinus* | 169 | ± 267 | 22% | 877 | ± 1205 | 274 | Genbank CP000581:CP000601 |
| *O. sativa* | 424 | ± 708 | 46% | 234 | ± 328 | 167 | Genbank NC_008394:NC_008405 |
| *P. falciparum* | 178 | ± 137 | 23% | 795 | ± 1602 | 312 | Genbank NC_000521, NC_000910, NC_004314:NC_004318, NC_004325:NC_004331 |
| *P. infestans* | 128 | ± 132 | 15% | 407 | ± 639 | 291 | Broad Institute [3] |
| *P. patens* | 306 | ± 724 | 52% | 226 | ± 258 | 180 | JGI [4] |
| *P. sojae* |  |  |  |  |  |  | None used |
| *P. tetraurelia* | 26 | ± 3 | 0% | 400 | ± 620 | 399 | Genbank CT867985:CT868681 |
| *P. trichocarpa* | 374 | ± 659 | 45% | 238 | ± 309 | 159 | v1.1 from DOE/JGI [4] |
| *P. tricornutum* | 131 | ± 238 | 12% | 857 | ± 819 | 561 | JGI [4] |
| *P. yoelii* | 208 | ± 277 | 24% | 662 | ± 1115 | 282 | Genbank |
| *R. oryzae* | 80 | ± 57 | 3% | 309 | ± 449 | 254 | Broad Institute [3] |
| *S. cerevisiae* | 315 | ± 459 | 42% | 1333 | ± 1156 | 125 | Genbank |
| *S. mansoni* | 2342 | ± 3192 | 80% | 217 | ± 236 | 207 | TIGR/JCVI [5] |
| *S. nodorum* | 83 | ± 75 | 6% | 441 | ± 586 | 397 | Broad Institute [3] |
| *S. pombe* | 83 | ± 69 | 6% | 587 | ± 826 | 263 | Genbank |
| *S. purpuratus* |  |  |  |  |  |  | None used |
| *S. sclerotiorum* | 141 | ± 130 | 22% | 388 | ± 574 | 355 | Broad Institute [3] |
| *T. rubripes* | 623 | ± 1927 | 40% | 163 | ± 194 | 146 | ENSEMBL release 45 [1, 2] |
| *T. thermophila* | 164 | ± 199 | 23% | 420 | ± 580 | 356 | TIGR/JCVI [5] |
| *U. maydis* | 128 | ± 110 | 17% | 1050 | ± 1241 | 297 | Broad Institute [3] |

^*^Calculated from all introns in the genome, from genome annotations.

^**^Fraction of all introns in the genome longer than 200 bp; calculated from genome annotations.

^§^Calculated from all exons in the genome, from genome annotations.

^†^Calculated from all internal exons in the genome (excluding first and last exons in a gene, and exons in genes without introns, which cannot be CEs), calculated from genome annotations.

**Supplementary Table S3 - Observed functional group enrichment of RIs is due primarily to highly expressed genes being more abundantly covered by our ESTs**

| Organism | Functional group | Type of group | # genes with RIs | # genes with EST coverage in functional group | # RIs in functional group | p-value |
| --- | --- | --- | --- | --- | --- | --- |
| *A. nidulans* | Carbohydrate metabolic process | GOSLIM | 57 | 85 | 12 | 7.3e-4 |
|  | Ribosome | GOSLIM | 57 | 69 | 10 | 3.7e-3 |
|  | Structural molecule activity | GOSLIM | 57 | 77 | 10 | 9.9e-3 |
|  | Catabolic process | GOSLIM | 57 | 66 | 9 | 1.6e-2 |
|  | Structural constituent of ribosome | GO | 57 | 69 | 10 | 3.4e-2 |
|  | Non-secretory | signalP | 57 | 1465 | 53 | 4.3e-2 |
| *M. grisea* | Ribosome | GOSLIM | 78 | 74 | 11 | 1.4e-3 |
|  | Structural molecule activity | GOSLIM | 78 | 81 | 11 | 3.4e-3 |
|  | Translation | GO | 78 | 85 | 12 | 7.7e-3 |
|  | Structural constituent of ribosome | GO | 78 | 73 | 11 | 9.9e-3 |
|  | Translation | GOSLIM | 78 | 117 | 13 | 5.6e-3 |
|  | Non-secretory | signalP | 78 | 1931 | 72 | 3.2e-3 |
| *S. sclerotiorum* | Ribosome | GOSLIM | 175 | 78 | 23 | 3.1e-10 |
|  | Structural constituent of ribosome | GO | 175 | 76 | 23 | 1.4e-9 |
|  | Structural molecule activity | GOSLIM | 175 | 84 | 23 | 1.7e-9 |
|  | Translation | GO | 175 | 84 | 23 | 1.5e-8 |
|  | Translation | GOSLIM | 175 | 126 | 26 | 7.5e-8 |
|  | Ribosome | GO | 175 | 62 | 14 | 2.2e-3 |

We examined functional group enrichment of RIs and CEs in seven organisms (*A. nidulans*, *C neoformans*, *C. immitis*, *N. crassa*, *M. grisea*, *S. sclerotiorum*, and *D. discoideum*). We used Blast2GO [6] to determine GO [7] categories, and used “generic” GOSLIM definitions to find GOSLIM categories using blast2GO.

We also used COG [8] categories in *A. nidulans* [9]. We also used the program signalP to determine whether proteins are secretory, non-secretory proteins, or membrane-anchored [10]. p-values are calculated from hypergeometric distribution and corrected with bonferroni correction. Bonferroni correction was calculated separately for each organism and for each type of functional group category (GO, GOslim, signalP, etc.). Only those functional groups with corrected p-values scoring < 0.05 are listed in this table.

We found significantly overrepresented categories in 3 of these 7 organisms (see Supplementary Information, Table S1). However, since the genes in which we observed RIs have higher-than-average EST coverage (including highly expressed genes such as genes related to the ribosome), it is likely that we are merely seeing a skewed subset of the highly expressed genes containing RIs. We also examined the CEs in *D. discoideum* and found no functional group overrepresentation.
